# A Single Shock During An Episode Of Ventricular Tachycardia: Is The Device Working Properly?

**DOI:** 10.1016/s0972-6292(16)30545-9

**Published:** 2012-09-01

**Authors:** Jesus Jimenez-Lopez, Miguel A Arias, Finn Akerstrom, Marta Pachon, Alberto Puchol

**Affiliations:** Cardiac Arrhythmia and Electrophysiology Unit, Department of Cardiology, Hospital Virgen de la Salud, Toledo, Spain

**Keywords:** Cardioverter-defibrillator, shock, evaluation

## Abstract

Emergency department admissions due to implantable cardioverter-defibrillator (ICD) shocks constitute an important patient group. The correct evaluation includes a review of system integrity and a careful analysis of stored intracardiac electrograms. We present a patient admitted with a single ICD discharge due to an episode of sustained monomorphic ventricular tachycardia, and an unexpected finding.

## Introduction

Inappropriate implantable cardioverter defibrillator (ICD) shocks are in most cases due to a supraventricular tachyarrhythmia misclassified by the device. Other causes of inappropriate discharges include T wave oversensing, atrial far-field sensing, double or triple sensing of ventricular signals, myopotentials, lead or connector block failure or electromagnetic interferences. A careful review of the stored intracardiac electrograms during the episodes usually identifies the underlying cause. However, in certain situations this may be difficult due to the presence of complex device programmed algorithms. Therefore a correct knowledge and recognition of these algorithms and stored episodes is critical for a proper understanding of the ICD function and when making subsequent management decisions. We report a patient who presented with a single ICD discharge due to a non-syncopal sustained monomorphic ventricular tachycardia, and an unexpected finding.

## Case Presentation

**Figure 1:**
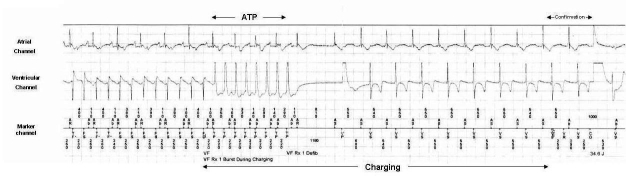
Ventricular tachycardia with a mean cycle length of 250 ms occurring within the VF zone, is initially treated by programmed ATP during charging, which correctly terminates the arrhythmia. At the end of charging, stable sinus rhythm can be identified. After VR, due to T wave oversensing, the next two RR intervals are incorrectly classified in VT detection interval + 60 ms range and a shock is delivered.

A 73-year-old man with a history of ischemic cardiomyopathy, severely reduced left ventricular ejection fraction and renal dysfunction, underwent a dual chamber ICD implant for secondary prevention. Five years later a system replacement (Maximo II DR D264DRM, Medtronic Inc., Minneapolis, MN, USA), due to a delayed device-related infection, was carried out. The new implanted device was programmed with three detection zones and corresponding therapies: ventricular tachycardia (VT-1) detection at 350 ms with antitachycardia pacing (ATP) and cardioversion (CV), fast ventricular tachycardia (VT-2) detection at 310 ms with ATP and CV and ventricular fibrillation (VF) detection at 260 ms with ATP during charging and defibrillation. Two months later, the patient was admitted to the emergency department due to a single nonsyncopal ICD shock. The stored intracardiac electrogram (Figure 1) showed a single arrhythmic episode classified as VF consisting of a regular monomorphic tachycardia with a mean cycle length of 250 ms with clearly different electrograms as compared with those in sinus rhythm, as well as atrio-ventricular dissociation, confirming true ventricular tachyarrhythmia. The ICD interrogation revealed no abnormal parameters for sensing and pacing. Antitachycardia pacing during charging correctly terminated the arrhythmia, achieving stable sinus rhythm. However, the device delivered a high-energy shock after completion of capacitors charging. Is the device functioning correctly?

## Discussion

Antitachycardia pacing during charging is a useful feature for reducing high-energy shocks delivered by an ICD when the patient suffer an episode of sustained ventricular tachycardia with a cycle length within VF zone. Programming this algorithm, ventricular tachycardia episodes longer than 240 ms, occurring within the VF zone, may be terminated successfully in 69% of cases [1], improving quality of life by decreasing the number of painful termination events [2]. ATP during charging is a programmable feature that consists of a burst (nominal) of eight (nominal) impulses at 88% (nominal) of the tachycardia cycle length. This therapy is applied while the capacitors are charging, when the device detects a ventricular tachycardia within the VF zone. If any one of the last eight RR intervals is shorter than programmed interval (nominal 240 ms), the device delivers a shock. By contrast, ATP is delivered if all of the last eight RR intervals are longer than or equal to the ATP cut off rate (nominal; 240 ms). Once the charge is enabled on the device after the delivery of the ATP, a confirmation window of the tachycardia is carried out by monitoring the ventricular rate for the next five beats. This confirmation works by analyzing cycle length for the beats after the charging is enabled (in contrast, if ATP during charging is not programmed on, confirmation window is carried out when the capacitors starts charging). If four of the last five analyzed beats have a cycle length longer than the VT-1 (or VF if VT-1 and fast ventricular tachycardia are off) detection interval plus 60 ms, shock is aborted. Otherwise, the device delivers a high-energy shock [3]. Certain delays during confirmation window up to shock have not been related with an increase of symptoms.

In the present case, a ventricular tachycardia with a cycle length of 250 ms is detected within the VF zone (Figure 1). The device applies a burst of eight impulses with a cycle length of 220 ms, with successful termination of the tachycardia. Subsequently, when the capacitor terminates to charge (Figure 1, CE on marker channel), though a stable sinus rhythm is observed in the electrograms, oversensing of the T wave results in two of the last three RR intervals falling within the VT-1 + 60 ms interval resulting in false confirmation of ongoing tachycardia and delivery of shock.

The first event after charging is enabled that is due to T wave oversensing and is sensed as ventricular refractory (Figure 1. VR), and is ignored. The next event is a true event followed again by oversensed T wave, thus the two intervals fall in the tachycardia detection interval + 60 ms range. The device assumes that the tachycardia is ongoing and subsequently delivers a shock.

New ICDs try to avoid this problem by an implementing algorithm called confirmation +. When the algorithm is programmed on the confirmation window starts just after ATP delivery. The rate considered for analysis is based on the cycle length of the detected arrhythmia, instead of the programmed cut-off zones. If four of the last five analyzed RR intervals have a cycle length longer than the cycle length of the detected tachycardia plus 60 ms, shock is aborted. The rhythm cycle length is calculated considering the six RR intervals immediately prior to detection, dropping the minimum and maximum intervals and taking the mean of the remaining four. Based on these criteria, the device classifies the rhythm as ongoing or terminated. Additional criteria may be applied depending on the detection zone and whether the rhythm is polymorphic.

On the other hand, possible causes for T-wave oversensing include diminution of R-wave amplitude and relative or dynamic gain in the T-wave amplitude, progressive cardiomyopathy, electrolyte abnormalities (such as hyperkalaemia), medications, injury current-related increase in the T-wave voltage, or changes in the sympathetic tone. Newer generation devices have an interesting feature called T-wave Discrimination that try to reduce the potential to deliver inappropriate therapy for high rates that are attributable to T-wave oversensing. When VT or VF is suspected, T-Wave Discrimination applies amplitude, rate, and pattern criteria to determine if both R-waves and T-waves are being sensed. If both are sensed, VT or VF detection is withheld. If only R-waves are sensed, VT or VF detection occurs. This algorithm is applied on initial detection and on redetection.

This case reflects the importance of a systematic review of the stored intracardiac electrograms and a thorough knowledge of the device programmed algorithms in patients with an ICD in order to correctly interpret the findings observed and to reprogramming device algorithms when needed.
